# Retraction Note: Agmatine protects retinal ganglion cells from hypoxia-induced apoptosis in transformed rat retinal ganglion cell line

**DOI:** 10.1186/s12868-023-00814-3

**Published:** 2023-08-14

**Authors:** Samin Hong, Jong Eun Lee, Chan Yun Kim, Gong Je Seong

**Affiliations:** 1 Institute of Vision Research, Department of Ophthalmology, Yonsei University College of Medicine, Seoul, Korea; 2 Department of Anatomy, Yonsei University College of Medicine, Seoul, Korea

**BMC Neuroscience volume (2007) 81**

The Editor has retracted this article. Overlap has been detected within the figures of this article, specifically:

- In Fig. 5A there appears to be overlap between the blots for the 3h pJNK and the 6h pJNK
- In Fig. 6A the NF-κB (Nucleus) 30min blot appears to overlap with the pNF-κB (cytoplasm) 30 min blot in Fig. 6B
- In Fig. 6A the pNF-κB (Nucleus) 1h blot appears to overlap with the NF-κB (cytoplasm) 1h blot in Fig. 6B
- In Fig. 6B the pNF-κB (cytoplasm) 1h blot appears to overlap with the pNF-κB (cytoplasm) 3h blot.

The Editor has therefore lost confidence in the integrity of the results presented in this article. None of the authors responded to any correspondence from the editor about this retraction.

